# Second Primary Malignancies in Patients With Melanoma Subtypes: Analysis of 120,299 Patients From the SEER Database (2000-2016)

**DOI:** 10.3389/fonc.2022.853076

**Published:** 2022-03-18

**Authors:** Asad Loya, Dan S. Gombos, Sapna P. Patel

**Affiliations:** ^1^ School of Medicine, Baylor College of Medicine, Houston, TX, United States; ^2^ Section of Ophthalmology, Department of Head and Neck Surgery, The University of Texas MD Anderson Cancer Center, Houston, TX, United States; ^3^ Department of Pediatrics, Division of Cancer Medicine, Baylor College of Medicine, Houston, TX, United States; ^4^ Department of Ophthalmology & Visual Sciences, The University of Texas Medical Branch at Galveston, Galveston, TX, United States; ^5^ Department of Melanoma Medical Oncology, The University of Texas MD Anderson Cancer Center, Houston, TX, United States

**Keywords:** second primary malignancies, melanoma, melanoma subtypes, uveal melanoma, non-acral cutaneous melanoma, acral lentiginous melanoma, mucosal melanoma, standardized incidence ratios

## Abstract

**Purpose:**

Evaluate incidence of second primary malignancies (SPM) after non-acral cutaneous melanoma (NACM), acral lentiginous melanoma (ALM), mucosal melanoma (MM), and uveal melanoma (UM).

**Patients and Methods:**

First primary NACM, ALM, MM, and UM cases diagnosed 2000-2016 were extracted from SEER. Seer*Stat was used to calculate excess absolute risks (EAR) and standardized incidence ratios (SIR) of SPMs relative to a matched cohort from the general population. P-value of 0.05 determined significance.

**Results:**

Inclusion criteria was met by 109,385 patients with NACM, 2166 with ALM, 2498 with MM, and 6250 with UM. Increased incidence of malignancies occurred after NACM (SIR 1.51; 95%CI, 1.49-1.54), ALM (SIR 1.59; 95%CI, 1.40-1.81), MM (SIR 2.14; 95%CI, 1.85-2.45), and UM (SIR 1.24; 95%CI, 1.14-1.34) relative to the general population. Cutaneous melanoma occurred more frequently after NACM (SIR 9.54; 95%CI, 9.27-9.83), ALM (SIR 12.19; 95%CI, 9.70-15.14), MM (SIR 10.05; 95%CI, 7.18-13.68), and UM (SIR 2.91; 95%CI, 2.27-3.66). Patients with initial NACM (SIR 2.44; 95%CI, 1.64-3.51) and UM (SIR 44.34; 95%CI, 29.91-63.29) demonstrated increased incidence of eye and orbit melanoma. Renal malignancies occurred more frequently after NACM (SIR 1.24; 95%CI, 1.11-1.38), MM (SIR 3.54; 95%CI, 1.62-6.72) and UM (SIR 1.68; 95%CI, 1.09-2.48). Increased incidence of thyroid malignancies was observed after NACM (SIR 1.83; 95%CI, 1.61-2.06), ALM (SIR 3.74; 95%CI, 1.71-7.11), MM (SIR 4.40; 95%CI, 1.77-9.06), and UM (SIR 3.79; 95%CI, 2.52-5.47). Increased incidence of lymphoma was observed after NACM (SIR 1.20; 95%CI, 1.09-1.31) and ALM (SIR 2.06; 95%CI, 1.13-3.46).

**Conclusion:**

Patients with NACM, ALM, MM, and UM have increased incidence of SPMs compared to that expected from the general population. Each of these melanoma subtypes had increased occurrence of cutaneous melanoma and thyroid cancer; some, but not all, had increased occurrence of renal malignancies, eye and orbit melanoma, and lymphoma.

## Background

Malignant melanoma, a serious and devastating disease, originates from melanocytes within the non-glabrous skin (non-acral cutaneous melanoma), palm and sole glabrous skin (acral lentiginous melanoma), mucosal membranes (mucosal melanoma), and the uvea (uveal melanoma) (1–3). Non-acral cutaneous melanoma (NACM) represents the most common subtype of melanoma, accounting for close to 90% of diagnoses; acral lentiginous melanoma (ALM), mucosal melanoma (MM), and uveal melanoma (UM) largely comprise the remainder of cases (4–6). Despite a shared cell origin, these subtypes differ greatly by genetic composition (1, 7), treatment response (8), and clinical outcomes (4, 6).

NACM generally portends a better prognosis than ALM, MM or UM, with 5-year survival rates of 91.3%, 80.3%, 34.0%, and 78.4% respectively (4, 6). If complete remission is attained, patient care becomes increasingly focused on surveillance for recurrences and management of cancer sequalae; second primary malignancies (SPMs) embody one such sequela. Although incidence of SPMs has been investigated for patients with cutaneous melanoma (CM; encompasses NACM and ALM) (9–11) and UM (12, 13), limited literature exists on SPMs specific to MM (14) and ALM (15). Prior study of SPMs associated with mucosal melanoma (14) are limited solely to those arising from the sinonasal cavity and prior study of SPMs associated with ALM (15) focuses on an exclusively Korean population; both lack site-specific SPM risk investigation. With gaps in current literature, consensus cancer guidelines provided by organizations such as National Comprehensive Cancer Network (NCCN) (16–18), Cancer Care Ontario (CCO) (19), Canadian Medical Association (CMA), and European Society for Medical Oncology (ESMO) (20) provide either no or very limited discussion on SPM risk and follow-up after these malignancies.

In order to address this literature gap, we conducted a retrospective analysis of the SEER database to evaluate if patients with NACM, ALM, MM, and UM demonstrate increased incidence for SPMs compared to the general population in the contemporary era (2000-2016). We performed additional analysis to identify specific sites and latency periods with elevated risk for secondary malignancies. We conduct, to the best of our knowledge, the first investigation of site-specific SPM risk after MM and ALM. National Cancer Institute’s (NCI) Surveillance, Epidemiology, and End Results (SEER) registries (21), a national population-based cancer database, has been used and validated for such analyses in the past (9–12, 22).

## Patients and Methods

### Data Source

Cases of melanoma were extracted from the SEER database, which is comprised of up to 21 cancer registries that geographically account for approximately 36.7% of the US population (21). The specific dataset used for this study, “Incidence - SEER 18 Regs excluding AK Research Data, Nov 2018 Sub (2000-2016)”, contained data from 18 registries with cases diagnosed between 2000 and 2016. The SEER program tracks incidence of new tumors and documents demographic, treatment, tumor, and survival data; however, it does not include behavioral risk factors (e.g. smoking, physical inactivity) and comorbid diseases. Institutional review board approval was not required for this study, as it utilized only deidentified data with permission from NCI.

### Data Collection

Patients diagnosed with NACM, ALM, MM, and UM between 2000-2016 were included in the study; cases that were not first primary malignancies, were diagnosed by death certificate, were diagnosed by autopsy record, or were of unknown age were excluded from analysis.

Cases of NACM were identified using International Classification of Diseases for Oncology third edition (ICD-O-3) morphological codes 8721/3-8743/3; 8745/3-8790/3 (malignant melanoma excluding malignant melanoma, NOS & acral lentiginous melanoma) and topographical codes C44.0-44.9 (skin). ALM was identified using morphological code 8744/3 (acral lentiginous melanoma) and topographical codes C44.6-C44.7 (skin of upper limb, shoulder, lower limb, and hip). MM cases were identified using morphological codes 8720/3-8790/3 (melanoma) and topographical codes C00.0–C06.9 (lip, tongue, gum, palate, mouth); C09.0–C14.8 (tonsil, oropharynx, nasopharynx, pyriform sinus, hypopharynx); C15.0–C16.9 (esophagus, stomach); C19.9–C21.8 (rectosigmoid junction, rectum, anus/anal canal); C30.0 (nasal cavity); C31.0–C31.9 (accessory sinuses); C51.0–C51.9 (vulva); C52.9–C53.9 (vagina, cervix uteri); C60.0–C63.9 (male genital organs); C64.9–C68.9 (urinary tract). Lastly, UM was identified using morphological codes 8720-8790 (melanoma) and topographical codes C69.2 (retina); C69.3 (choroid); C69.4 (ciliary body, iris). “Retinal” melanomas (0.9%; 56/6250) were included as they most likely represent misclassification of uveal melanoma, a phenomenon described in previous studies (12, 23). ICD-O-3 codes used to identify NACM (6), ALM (6), MM (4), and UM (23) were consistent with prior studies investigating these malignancies. Patient demographics collected included age at diagnosis, race, and sex. Tumor data included laterality, histology, and site of origin.

### Statistical Analysis

Demographic and tumor data was tabulated. The multiple primary standardized incidence ratio (MP-SIR) algorithm of the Seer*stat program (version 8.3.6.1) was used to obtain standardized incidence ratios (SIR) and excess absolute risk (EAR) for second primary malignancies in patients with NACM, ALM, MM, and UM compared to a reference group representative of the general population, with similar sex, race (white/unknown, black, other) age-group (5-year interval), and calendar year of diagnosis (5-year interval). The algorithm was then further used to identify specific latency periods in which there was increased incidence of SPMs relative to the reference population. The authors (AL, SP, DSG) examined the site-specific analysis to identify trends across melanoma subtypes.

Analysis was limited to second malignancies only (early exit at next malignancy) to isolate relationship of subsequent malignancies with the first primary. Only malignant neoplasms diagnosed greater than two months after the melanoma diagnosis were considered to be second primaries, in order to distinguish them from concurrent malignancies discovered during screening. The reference population linked to the SEER database is comprised of Census Bureau data, through partnership with the National Center for Health Statistics (https://seer.cancer.gov/popdata/). An alpha level of significance of 0.05 was used for the study, and EAR was calculated per 10,000 individuals. IBM Statistical Product and Service Solutions (SPSS) version 26 and Microsoft Excel version 16.38 were used to conduct descriptive analysis and generate charts.

## Results

### Baseline Characteristics

Inclusion criteria was met by 109,385 patients with NACM, 2166 patients with ALM, 2498 patients with MM, and 6250 patients with UM for a total of 120,299 patients. The median (+/- SD) follow-up period for patients with NACM was 5.6 (+/- 4.7) years, with ALM was 4.3 (+/- 4.5) years, with MM was 1.7 (+/- 3.6) years, and with UM was 4.8 (+/- 4.4) years. During this period 11.4% (12472/109,385) of NACM patients, 10.8% (235/2166) of ALM patients, 8.1% (203/2498) of MM patients, and 9.4% (586/6250) of UM patients developed SPMs.

Most patients with initial NACM (56.1%; 61,385/109,385) and UM (52.4%; 3274/6250) were male, whereas most patients with initial ALM (55.4%; 1201/2166) and MM (71.8%; 1793/2498) were female. Majority of patients with NACM (94.5%;103,390/109,385), ALM (81.7%; 1770/2166), MM (84.9%; 2120/2498) and UM (96.2%; 6012/6250) were white. Almost all diagnoses of initial NACM (99.9%; 109,285/109,385), ALM (100%; 2166/2166), and MM (99.8%; 2493/2498) were microscopically confirmed whereas only 53.8% (3365/6250) of UM cases were microscopically confirmed. Additional patient characteristics are displayed in **Table 1**.

**Table 1:** Demographic, tumor, and treatment characteristics of patients with first primary melanoma.

		Non-Acral Cutaneous Melanoma				Acral Lentiginous Melanoma				Mucosal Melanoma				Uveal Melanoma			
		Overall Cohort (n=109,385)		SPM Cohort (n=12472)		Overall Cohort (n=2166)		SPM Cohort (n=235)		Overall Cohort (n=2498)		SPM Cohort (n=203)		Overall Cohort (n=6250)		SPM Cohort (n=586)	
		Count	N %	Count	N %	Count	N %	Count	N %	Count	N %	Count	N %	Count	N %	Count	N %
Age-groups	0-49 years	32956	30.1%	1941	15.6%	480	22.2%	21	8.9%	342	13.7%	21	10.3%	1333	21.3%	52	8.9%
	50-64 years	35988	32.9%	4238	34.0%	670	30.9%	70	29.8%	698	27.9%	61	30.0%	2353	37.6%	234	39.9%
	65+ years	40441	37.0%	6293	50.5%	1016	46.9%	144	61.3%	1458	58.4%	121	59.6%	2564	41.0%	300	51.2%
Sex	Female	48000	43.9%	4194	33.6%	1201	55.4%	110	46.8%	1793	71.8%	148	72.9%	2976	47.6%	247	42.2%
	Male	61385	56.1%	8278	66.4%	965	44.6%	125	53.2%	705	28.2%	55	27.1%	3274	52.4%	339	57.8%
Race	White	103390	94.5%	12307	98.7%	1770	81.7%	193	82.1%	2120	84.9%	179	88.2%	6012	96.2%	574	98.0%
	Black	323	0.3%	43	0.3%	185	8.5%	19	8.1%	139	5.6%	8	3.9%	54	0.9%	3	0.5%
	Asian/Pacific Islander	611	0.6%	46	0.4%	170	7.8%	22	9.4%	208	8.3%	16	7.9%	74	1.2%	5	0.9%
	American Indian/Alaska Native	210	0.2%	14	0.1%	12	0.6%	0	0.0%	17	0.7%	0	0.0%	16	0.3%	3	0.5%
	Unknown	4851	4.4%	62	0.5%	29	1.3%	1	0.4%	14	0.6%	0	0.0%	94	1.5%	1	0.2%
Diagnostic Confirmation	Microscopic	109285	99.9%	12469	100.0%	2166	100.0%	235	100.0%	2493	99.8%	201	99.0%	3365	53.8%	310	52.9%
	Not microscopic	15	0.0%	3	0.0%	0	0.0%	0	0.0%	4	0.2%	2	1.0%	2800	44.8%	270	46.1%
	Unknown	85	0.1%	0	0.0%	0	0.0%	0	0.0%	1	0.0%	0	0.0%	85	1.4%	6	1.0%
Summary stage	Carcinoma *In situ*	0	0.0%	0	0.0%	0	0.0%	0	0.0%	0	0.0%	0	0.0%	0	0.0%	0	0.0%
	Localized	95812	87.6%	11050	88.6%	1471	67.9%	163	69.4%	676	27.1%	83	40.9%	0	0.0%	0	0.0%
	Regional	10209	9.3%	1147	9.2%	582	26.9%	63	26.8%	337	13.5%	22	10.8%	0	0.0%	0	0.0%
	Distant	1607	1.5%	99	0.8%	85	3.9%	6	2.6%	241	9.6%	10	4.9%	0	0.0%	0	0.0%
	Unknown/unstaged	1757	1.6%	176	1.4%	28	1.3%	3	1.3%	1244	49.8%	88	43.3%	6250	100.0%	586	100.0%

SPM, second primary malignancy.

### SPM Incidence

**Figure 1:**
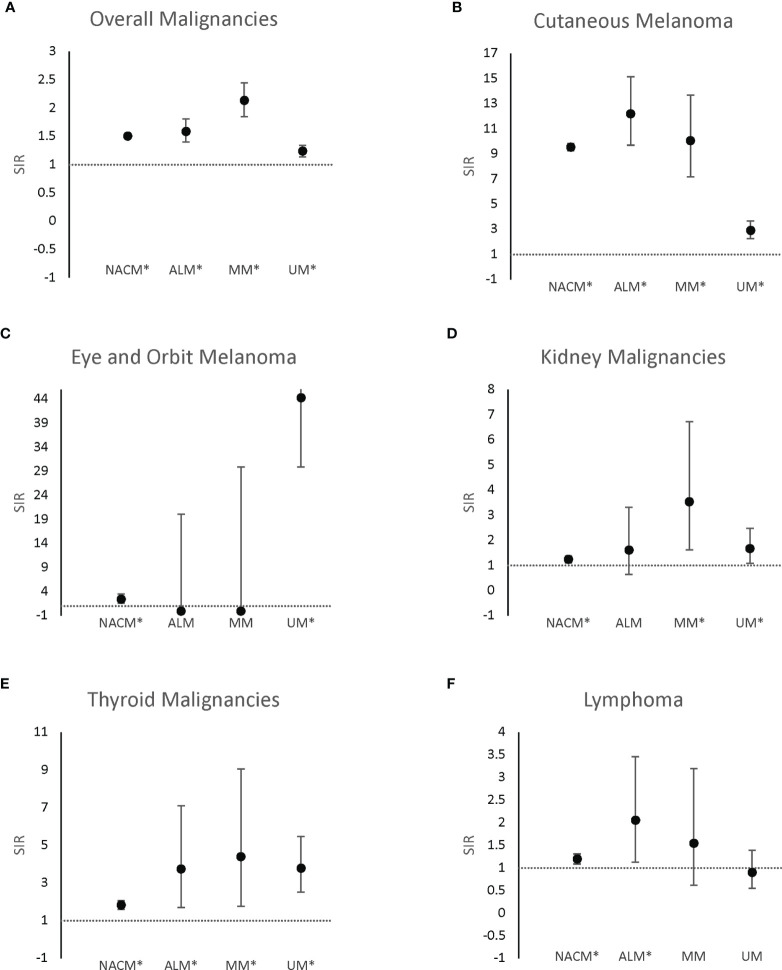
Standardized incidence ratios of secondary malignancies grouped by category. Standardized incidence ratios of overall secondary malignancies **(A)**, secondary cutaneous melanoma **(B)**, secondary eye and orbit melanoma **(C)**, secondary kidney malignancies **(D)**, secondary thyroid malignancies **(E)**, and secondary lymphoma **(F)** following first primary melanomas. ALM, acral lentiginous melanoma; MM, mucosal melanoma, NACM, non-acral cutaneous melanoma; UM, uveal melanoma. *p<0.05.

Relative to the general population, an increased incidence of new malignancies was observed in patients with initial NACM (SIR 1.51; 95% CI, 1.49 to 1.54; EAR 64.46), ALM (SIR 1.59; 95% CI, 1.40 to 1.81; EAR 79.56), MM (SIR 2.14; 95% CI, 1.85 to 2.45; EAR 153.59), and UM (SIR 1.24; 95% CI, 1.14 to 1.34; EAR 33.04). Notably, increased incidence of secondary CM, eye and orbit melanoma, kidney cancer, thyroid cancer, and lymphoma were observed across some melanoma subtypes (**Table 2** and **Figure 1**).

**Table 2:** Notable second primary malignancies by specific site following first primary melanoma.

	NACM (n=109,385)		ALM (n=2166)		MM (n=2498)		UM (n=6250)	
	O/E (95%CI)	EAR^a^	O/E (95%CI)	EAR^a^	O/E (95%CI)	EAR^a^	O/E (95%CI)	EAR^a^
All Sites	1.51* (1.49-1.54)	64	1.59* (1.4-1.81)	80	2.14* (1.85-2.45)	154	1.24* (1.14-1.34)	33
Melanoma of the Skin	9.54* (9.27-9.83)	61	12.19* (9.7-15.14)	68	10.05* (7.18-13.68)	51	2.91* (2.27-3.66)	14
Kidney	1.24* (1.11-1.38)	1	1.61 (0.65-3.32)	2	3.54* (1.62-6.72)	9	1.68* (1.09-2.48)	3
Eye and Orbit - Melanoma	2.44* (1.64-3.51)	0	0 (0-20.09)	0	0 (0-29.94)	0	44.34* (29.91-63.29)	9
Thyroid	1.83* (1.61-2.06)	2	3.74* (1.71-7.11)	6	4.40* (1.77-9.06)	8	3.79* (2.52-5.47)	6
Lymphoma	1.20* (1.09-1.31)	1	2.06* (1.13-3.46)	7	1.55 (0.62-3.2)	4	0.9 (0.55-1.39)	-1

ALM, acral lentiginous melanomaI, confidence interval; E, expected; EAR, excess absolute risk; MM, mucosal melanoma; NACM, non-acral cutaneous melanoma; O, observed, SIR, standardized incidence ratio; UM, uveal melanoma.

*P < 0.05.

a Excess absolute risk is per 10,000.

CM occurred more frequently in patients with initial NACM (SIR 9.54; 95% CI, 9.27 to 9.83; EAR 60.72), ALM (SIR 12.19; 95% CI, 9.70 to 15.14; EAR 68.41), MM (SIR 10.05; 95% CI, 7.18 to 13.68; EAR 51.23), and UM (SIR 2.91; 95% CI, 2.27 to 3.66; EAR 13.75) than expected from the general population. On the other hand, only patients with initial NACM (SIR 2.44; 95% CI, 1.64 to 3.51; EAR 0.26) and UM (SIR 44.34; 95% CI, 29.91 to 63.29; EAR 8.54) demonstrated increased incidence of eye and orbit melanoma, whereas patients with ALM (SIR 0.00; 95% CI, 0.00 to 20.09; EAR -0.17) and MM (SIR 0.00; 95% CI, 0.00 to 29.94; EAR -0.18) demonstrated no significant difference from the reference population. Renal malignancies were noted to occur more frequently in patients with NACM (SIR 1.24; 95% CI, 1.11 to 1.38; EAR 0.96), MM (SIR 3.54; 95% CI, 1.62 to 6.72; EAR 9.19) and UM (SIR 1.68; 95% CI, 1.09 to 2.48; EAR 2.95), but not ALM (SIR 1.61; 95% CI, 0.65 to 3.32; EAR 2.41). Increased incidence of thyroid malignancies was observed in patients with initial NACM (SIR 1.83; 95% CI, 1.61 to 2.06; EAR 1.79), ALM (SIR 3.74; 95% CI, 1.71 to 7.11; EAR 5.99), MM (SIR 4.40; 95% CI, 1.77 to 9.06; EAR 7.69), and UM (SIR 3.79; 95% CI, 2.52 to 5.47; EAR 6.00). Lastly, increased incidence of lymphoma was observed in patients with initial NACM (SIR 1.20; 95% CI, 1.09 to 1.31; EAR 1.17) and ALM (SIR 2.06; 95% CI, 1.13 to 3.46; EAR 6.55) but not MM (SIR 1.55; 95% CI, 0.62 to 3.20; EAR 3.55) or UM (SIR 0.90; 95% CI, 0.55 to 1.39; EAR -0.63).

### SPM Latency Analysis

Patients with NACM demonstrated elevated incidence of overall SPMs during the first year (2-11 months) following diagnosis (SIR 2.12; 95% CI 2.03 to 2.21), 1-5 years following diagnosis (SIR 1.57; 95% CI 1.53 to 1.61), 5-10 years following diagnosis (SIR 1.30; 95% CI 1.25 to 1.34), and greater than 10 years following diagnosis (SIR 1.21; 95% CI 1.15 to 1.28). Similarly, patients with MM had increased incidence of overall SPMs during the first year (2-11 months) following diagnosis (SIR 2.38; 95% CI 1.81 to 3.08), 1-5 years following diagnosis (SIR 2.12; 95% CI 1.72 to 2.58), 5-10 years following diagnosis (SIR 1.81; 95% CI 1.24 to 2.56), and greater than 10 years following diagnosis (SIR 2.24; 95% CI 1.23 to 3.76). In contrast, those with ALM only had increased incidence of overall SPMs the first year following diagnosis (SIR 1.98; 95% CI 1.45 to 2.66) and 1-5 years following diagnosis (SIR 1.72; 95% CI 1.43 to 2.06). Those with UM demonstrated elevated incidence of overall SPMs during the first year following diagnosis (SIR 1.53; 95% CI 1.24 to 1.86; EAR 69.12), 1-5 years following diagnosis (SIR 1.21; 95% CI 1.07 to 1.37; EAR 28.62), and 5-10 years following diagnosis (SIR 1.20; 95% CI 1.02 to 1.39; EAR 27.78). High-risk latency periods further differed by SPM types (**Table 3** and **Supplementary Material**).

**Table 3:** Risk of second primary malignancy distributed by time from diagnosis of first primary malignancy.

		2-11 months	12-59 months	60-119 months	120+ months
		SIR (95%CI)	SIR (95%CI)	SIR (95%CI)	SIR (95%CI)
Non-Acral Cutaneous Melanoma	All Sites	2.12* (2.03-2.21)	1.57* (1.53-1.61)	1.30* (1.25-1.34)	1.21* (1.15-1.28)
	Melanoma of the Skin	16.94* (15.9-18.04)	10.45* (10.01-10.9)	7.03* (6.6-7.47)	5.68* (5.11-6.29)
	Kidney	2.04* (1.59-2.59)	1.16 (0.97-1.37)	0.94 (0.74-1.18)	1.41* (1.04-1.86)
	Eye and Orbit - Melanoma	0.67 (0.02-3.72)	3.26* (1.9-5.23)	2.50* (1.14-4.75)	1.29 (0.16-4.65)
	Thyroid	4.71* (3.72-5.87)	1.82* (1.49-2.19)	1.15 (0.86-1.5)	1 (0.61-1.54)
	Lymphoma	2.22* (1.83-2.68)	1.12 (0.97-1.29)	0.9 (0.74-1.09)	1.14 (0.86-1.47)
Acral Lentiginous Melanoma	All Sites	1.98* (1.45-2.66)	1.72* (1.43-2.06)	1.29 (0.96-1.7)	1.21 (0.73-1.9)
	Melanoma of the Skin	14.45* (7.9-24.24)	14.95* (10.94-19.94)	8.49* (4.85-13.79)	7.56* (2.77-16.45)
	Thyroid	5.96 (0.72-21.55)	5.47* (2.01-11.9)	1.43 (0.04-7.96)	0 (0-13.6)
	Lymphoma	5.88* (2.16-12.8)	1.88 (0.69-4.09)	1.09 (0.13-3.92)	0 (0-5.02)
Mucosal Melanoma	All Sites	2.38* (1.81-3.08)	2.12* (1.72-2.58)	1.81* (1.24-2.56)	2.24* (1.23-3.76)
	Melanoma of the Skin	10.37* (4.97-19.08)	13.01* (8.42-19.2)	3.76 (0.77-10.98)	6.73 (0.82-24.32)
	Kidney	7.79* (2.53-18.17)	1.6 (0.19-5.77)	2.11 (0.05-11.73)	5.76 (0.15-32.09)
	Thyroid	10.98* (2.99-28.12)	3.94 (0.81-11.52)	0 (0-11.1)	0 (0-27.61)
Uveal Melanoma	All Sites	1.53* (1.24-1.86)	1.21* (1.07-1.37)	1.20* (1.02-1.39)	1.12 (0.85-1.44)
	Melanoma of the Skin	4.41* (2.41-7.39)	2.68* (1.81-3.82)	2.58* (1.55-4.03)	2.97* (1.36-5.63)
	Kidney	4.58* (2.09-8.69)	1.31 (0.6-2.5)	1.15 (0.37-2.68)	1.18 (0.14-4.27)
	Eye and Orbit - Melanoma	32.63* (6.73-95.36)	44.80* (24.49-75.16)	66.25* (35.27-113.28)	0 (0-48.57)
	Thyroid	11.20* (5.59-20.04)	3.80* (2.02-6.49)	0.46 (0.01-2.56)	3.68 (0.76-10.77)

CI, confidence interval; SIR, standardized incidence ratio.

*P < 0.05.

## Discussion

Using a national cancer database, we analyzed 120,299 patients with various melanoma subtypes and found an elevated incidence of SPMs relative to the general population. Notably, all four melanoma subtypes (NACM, ALM, MM, UM) demonstrated increased risk of secondary CM and thyroid cancer, and some but not all melanoma subtypes demonstrated increased risk for secondary renal malignancies (NACM, MM, UM), eye and orbit melanoma (NACM, UM), and lymphoma (NACM, ALM).

A biologic rationale exists for the findings in our study. CM and thyroid cancers commonly harbor oncogenic mutations of the mitogen-activated protein kinase (MAPK) pathway (24–29). Renal cancers share immunogenicity and *BAP1* aberrations with CM and UM (30–35). Lymphomas and melanomas are associated with decreased immune surveillance (36–43).

Although historic and smaller retrospective analyses of the SEER database have examined SPMs following CM (9, 10) and UM (12), herein we provide, to the best of our knowledge, the first investigation of site-specific SPM risk after MM and ALM. Moreover, through analysis of UM in a larger and more contemporary cohort, we highlight increased incidence of secondary thyroid malignancies, a finding undetected in prior investigation (12). Bradford, et al. (9) and Spanogle, et al. (10) investigated incidence of SPMs following CM in various subsets of the SEER database and both found increased incidence of secondary CM, eye and orbit melanoma, thyroid cancer, renal cancer, and lymphoma. Similarly, Vakharia, et al. (11) in their investigation of secondary malignancies excluding CM demonstrated increased risk for these sites. Their findings (9–11) were consistent with our findings of SPMs following NACM. Laíns, et al. (12) investigated risk of second primary malignancies following UM and found increased incidence for secondary CM, eye and orbit melanoma, and renal cancer but not a significant increase in thyroid cancer. However, the study (12) showed a strong trend of increased thyroid cancer (SIR 2.06, 95% CI 0.99 to 3.78) in a cohort of 3976 patients, which with increased power may have captured a significant result similar to our study.

Despite a growing body of literature on SPMs, national consensus guidelines such as NCCN (16–18) (US), Canada CCO (19) (Canada), CMA (Canada) and ESMO (20) (Europe) sparsely address secondary malignancies in their follow-up recommendations. Canadian (19) and European (20) guidelines discuss only an increased risk for secondary cutaneous malignancies after initial CM and the importance of long-term dermatological surveillance; these guidelines lack SPM follow-up recommendations specific to ALM, MM, and UM. American consensus guidelines provide slightly more insight on non-cutaneous SPMs following CM by discussing the role of genetic testing in determining SPM risk and by providing guidance on when to consider such testing (16). However, American guidelines do not remark on the increased incidence of lymphoma or thyroid cancer (16). Moreover, these guidelines state that CM is not associated with an increased risk for UM (17).

Indeed, developing follow-up recommendations poses a challenge as cost, clinical benefit, and burden of increased health-care visits must all be balanced. Nonetheless, increased awareness of the associations studied herein are paramount to guiding appropriate clinician judgement when caring for melanoma patients; discussion of up-to-date evidence in national guidelines can improve patient care and long-term health outcomes. With a more appropriate index of suspicion, lesions (e.g. renal cyst, thyroid nodule) and atypical findings discovered during diagnostic or surveillance imaging that may otherwise have been dismissed as benign may instead be deemed to warrant additional follow-up. Furthermore, symptoms concerning for an associated SPM (e.g. visual flashers and floaters) can be interpreted more appropriately and potentially lead to earlier diagnosis. The high-risk latency periods identified by this study may provide additional clinical insight when deciding further management for patients presenting with these signs or symptoms.

The authors propose that cost effective screening such as total body skin exams be recommended for patients with all subtypes (NACM, ALM, MM, UM) of melanoma; prior studies (44, 45) support the economic efficiency of targeted screening strategies in high-risk groups. The authors suggest that patients with NACM should receive routine complete eye exams (including dilated fundus exam) at least as often as recommend for asymptomatic adults without risk factors for ocular disease by American Academy of Ophthalmology (AAO) guidelines (46): every 5 to 10 years when less than 40 years of age, every 2 to 4 years when between 40 and 54 years of age, every 1 to 3 years when between 55 and 64 years of age, and every 1 to 2 years when 65 or more years of age. Surveillance for SPM in patients undergoing screening measures versus those who do not undergo these screening exams may further elucidate the role and feasibility of monitoring for the development of cancer in these patients. Moreover, it would be prudent to identify high-risk groups and factors through prediction models for subsequent melanoma, such as those described by Cust et al. (47), to help guide effective recommendations.

### Limitations in the Study Design

An important limitation of the SEER database and our study is the possibility of miscoding a recurrence as a second primary malignancy when pathologic evaluation is unavailable and tumor location is identical. This most directly impacts the calculation of secondary eye and orbit melanoma after initial UM (6.67% cases without microscopic confirmation), as UM is largely diagnosed clinically through examination and imaging rather than pathologically (48); indeed, this finding is more likely representative of recurrences rather than true SPMs. Another important limitation is the inability of Seer.Stat’s MP-SIR algorithm to analyze site-specific secondary malignancy incidence beyond those preset in the software. As a result, we were unable to provide analysis on incidence of NACM and ALM as secondary malignancies, and instead had them grouped as secondary CM. Other limitations include other possible miscoding and inability to account for variables not included within the database. Additionally, patients that moved to a geographical area not covered by SEER could be lost to follow-up leading to underreporting. Despite these limitations, however, the national database has been validated for SPM analyses (9–12, 22).

## Conclusions

We found patients with NACM, ALM, MM, and UM to have increased incidence of SPMs compared to that expected from the general population. Each of these melanoma subtypes had increased occurrence of secondary CM and thyroid cancer; some, but not all, had increased occurrence of secondary renal malignancies, eye and orbit melanoma, and lymphoma. These patients may benefit from cost-effective screening methods such as full body skin exams. Patients with NACM should, at a minimum, receive age-appropriate comprehensive eye screening per national guidelines. Increased awareness of these associations is prudent to guiding clinical follow-up and additional studies are necessary to identify best-practice screening guidelines.

## Data Availability Statement

The original contributions presented in the study are included in the article/**Supplementary Material**. Further inquiries can be directed to the corresponding author.

## Author Contributions

Conception: SP. Study design: AL, DG, and SP. Statistical analysis: AL. Data interpretation: AL, DG, and SP. Manuscript preparation: AL, DG, and SP. All authors contributed to the article and approved the submitted version.

## Conflict of Interest

The authors declare that the research was conducted in the absence of any commercial or financial relationships that could be construed as a potential conflict of interest.

## Publisher’s Note

All claims expressed in this article are solely those of the authors and do not necessarily represent those of their affiliated organizations, or those of the publisher, the editors and the reviewers. Any product that may be evaluated in this article, or claim that may be made by its manufacturer, is not guaranteed or endorsed by the publisher.
