# Supplementary material for: Second Primary Malignancies in Patients With Melanoma Subtypes: Analysis of 120,299 Patients From the SEER Database (2000-2016)
Source: Front Oncol. 2022 Mar 18;12:853076. doi: 10.3389/fonc.2022.853076 (PMC8972193; doi:10.3389/fonc.2022.853076)
Supplement: Supplementary file 1 [file Table_1.docx]

| *Supplementary Table 1: Second Primary Malignancies by Specific Site Following First Primary Non-acral Cutaneous Melanoma* | | | | | | |
| --- | --- | --- | --- | --- | --- | --- |
|  | O | E | O/E | 95% CI LB | 95% CI UB | EAR^a^ |
| All Sites | 12,472 | 8,237.52 | 1.51* | 1.49 | 1.54 | 64.46 |
| All Solid Tumors | 11,246 | 7,269.61 | 1.55* | 1.52 | 1.58 | 60.53 |
| Oral Cavity and Pharynx | 204 | 214.31 | 0.95 | 0.83 | 1.09 | -0.16 |
| Lip | 25 | 17.04 | 1.47 | 0.95 | 2.17 | 0.12 |
| Tongue | 48 | 66.02 | 0.73* | 0.54 | 0.96 | -0.27 |
| Salivary Gland | 48 | 24.55 | 1.96* | 1.44 | 2.59 | 0.36 |
| Floor of Mouth, and Gum and Other Mouth | 24 | 39.17 | 0.61* | 0.39 | 0.91 | -0.23 |
| Floor of Mouth | 3 | 10.49 | 0.29* | 0.06 | 0.84 | -0.11 |
| Gum and Other Mouth | 21 | 28.68 | 0.73 | 0.45 | 1.12 | -0.12 |
| Tonsil | 38 | 35.52 | 1.07 | 0.76 | 1.47 | 0.04 |
| Pharynx | 14 | 27 | 0.52* | 0.28 | 0.87 | -0.2 |
| Nasopharynx | 3 | 6.29 | 0.48 | 0.1 | 1.39 | -0.05 |
| Oropharynx | 5 | 8.33 | 0.6 | 0.19 | 1.4 | -0.05 |
| Hypopharynx | 6 | 12.38 | 0.48 | 0.18 | 1.05 | -0.1 |
| Other Oral Cavity and Pharynx | 7 | 5 | 1.4 | 0.56 | 2.88 | 0.03 |
| Digestive System | 1,240 | 1,522.97 | 0.81* | 0.77 | 0.86 | -4.31 |
| Esophagus | 78 | 97.56 | 0.80* | 0.63 | 1 | -0.3 |
| Stomach | 89 | 128.87 | 0.69* | 0.55 | 0.85 | -0.61 |
| Small Intestine | 36 | 38.56 | 0.93 | 0.65 | 1.29 | -0.04 |
| Colon, Rectum and Anus | 659 | 792.05 | 0.83* | 0.77 | 0.9 | -2.03 |
| Colon and Rectum | 628 | 762.36 | 0.82* | 0.76 | 0.89 | -2.05 |
| Colon excluding Rectum | 467 | 548.89 | 0.85* | 0.78 | 0.93 | -1.25 |
| Cecum | 113 | 122.84 | 0.92 | 0.76 | 1.11 | -0.15 |
| Appendix | 27 | 13.32 | 2.03* | 1.34 | 2.95 | 0.21 |
| Ascending Colon | 97 | 108.07 | 0.9 | 0.73 | 1.09 | -0.17 |
| Hepatic Flexure | 24 | 27.76 | 0.86 | 0.55 | 1.29 | -0.06 |
| Transverse Colon | 41 | 52.98 | 0.77 | 0.56 | 1.05 | -0.18 |
| Splenic Flexure | 10 | 16.57 | 0.6 | 0.29 | 1.11 | -0.1 |
| Descending Colon | 25 | 30.69 | 0.81 | 0.53 | 1.2 | -0.09 |
| Sigmoid Colon | 107 | 140.58 | 0.76* | 0.62 | 0.92 | -0.51 |
| Large Intestine, NOS | 23 | 36.08 | 0.64* | 0.4 | 0.96 | -0.2 |
| Rectum and Rectosigmoid Junction | 161 | 213.46 | 0.75* | 0.64 | 0.88 | -0.8 |
| Rectosigmoid Junction | 42 | 54.19 | 0.78 | 0.56 | 1.05 | -0.19 |
| Rectum | 119 | 159.27 | 0.75* | 0.62 | 0.89 | -0.61 |
| Anus, Anal Canal and Anorectum | 31 | 29.69 | 1.04 | 0.71 | 1.48 | 0.02 |
| Rectum, Rectosig Junct, Anus, Anal Canal and Anorectum | 192 | 243.15 | 0.79* | 0.68 | 0.91 | -0.78 |
| Liver, Gallbladder, Intrahep Bile Duct and Other Biliary | 119 | 200.25 | 0.59* | 0.49 | 0.71 | -1.24 |
| Liver | 71 | 127.6 | 0.56* | 0.43 | 0.7 | -0.86 |
| Gallbladder | 4 | 19.27 | 0.21* | 0.06 | 0.53 | -0.23 |
| Intrahep and Extrahep Bile Ducts, and Other Biliary | 44 | 53.38 | 0.82 | 0.6 | 1.11 | -0.14 |
| Pancreas | 242 | 239.21 | 1.01 | 0.89 | 1.15 | 0.04 |
| Retroperitoneum | 5 | 5.78 | 0.86 | 0.28 | 2.02 | -0.01 |
| Peritoneum, Omentum and Mesentery | 4 | 8.47 | 0.47 | 0.13 | 1.21 | -0.07 |
| Other Digestive Organs | 8 | 12.22 | 0.65 | 0.28 | 1.29 | -0.06 |
| Skin excluding Basal and Squamous | 4,583 | 511.03 | 8.97* | 8.71 | 9.23 | 61.98 |
| Melanoma of the Skin | 4,456 | 466.84 | 9.54* | 9.27 | 9.83 | 60.72 |
| Other Non-Epithelial Skin | 127 | 44.19 | 2.87* | 2.4 | 3.42 | 1.26 |
| Breast | 891 | 854.8 | 1.04 | 0.98 | 1.11 | 0.55 |
| Female Genital System | 282 | 329.49 | 0.86* | 0.76 | 0.96 | -0.72 |
| Cervix Uteri | 20 | 35.59 | 0.56* | 0.34 | 0.87 | -0.24 |
| Corpus and Uterus, NOS | 166 | 178.99 | 0.93 | 0.79 | 1.08 | -0.2 |
| Ovary | 77 | 82.47 | 0.93 | 0.74 | 1.17 | -0.08 |
| Vagina | 3 | 5.06 | 0.59 | 0.12 | 1.73 | -0.03 |
| Vulva | 13 | 19.04 | 0.68 | 0.36 | 1.17 | -0.09 |
| Other Female Genital Organs | 3 | 8.34 | 0.36 | 0.07 | 1.05 | -0.08 |
| Male Genital System | 1,799 | 1,464.85 | 1.23* | 1.17 | 1.29 | 5.09 |
| Prostate | 1,765 | 1,438.56 | 1.23* | 1.17 | 1.29 | 4.97 |
| Testis | 15 | 14.13 | 1.06 | 0.59 | 1.75 | 0.01 |
| Penis | 12 | 9.46 | 1.27 | 0.66 | 2.22 | 0.04 |
| Other Male Genital Organs | 7 | 2.7 | 2.59* | 1.04 | 5.34 | 0.07 |
| Urinary System | 803 | 820.31 | 0.98 | 0.91 | 1.05 | -0.26 |
| Urinary Bladder | 444 | 513.76 | 0.86* | 0.79 | 0.95 | -1.06 |
| Kidney | 328 | 265.21 | 1.24* | 1.11 | 1.38 | 0.96 |
| Renal Pelvis | 13 | 20.17 | 0.64 | 0.34 | 1.1 | -0.11 |
| Ureter | 9 | 12.99 | 0.69 | 0.32 | 1.31 | -0.06 |
| Other Urinary Organs | 9 | 8.19 | 1.1 | 0.5 | 2.09 | 0.01 |
| Eye and Orbit | 32 | 14.9 | 2.15* | 1.47 | 3.03 | 0.26 |
| Eye and Orbit - Non-Melanoma | 3 | 3.04 | 0.99 | 0.2 | 2.88 | 0 |
| Eye and Orbit - Melanoma | 29 | 11.86 | 2.44* | 1.64 | 3.51 | 0.26 |
| Brain and Other Nervous System | 120 | 92.65 | 1.30* | 1.07 | 1.55 | 0.42 |
| Brain | 114 | 88.88 | 1.28* | 1.06 | 1.54 | 0.38 |
| Cranial Nerves Other Nervous System | 6 | 3.77 | 1.59 | 0.58 | 3.46 | 0.03 |
| Endocrine System | 271 | 150.44 | 1.80* | 1.59 | 2.03 | 1.84 |
| Thyroid | 260 | 142.46 | 1.83* | 1.61 | 2.06 | 1.79 |
| Thymus, Adrenal Gland and Other Endocrine | 11 | 7.99 | 1.38 | 0.69 | 2.46 | 0.05 |
| All Lymphatic and Hematopoietic Diseases | 912 | 752.57 | 1.21* | 1.13 | 1.29 | 2.43 |
| Lymphoma | 465 | 388.2 | 1.20* | 1.09 | 1.31 | 1.17 |
| Hodgkin Lymphoma | 23 | 23.13 | 0.99 | 0.63 | 1.49 | 0 |
| Non-Hodgkin Lymphoma | 442 | 365.07 | 1.21* | 1.1 | 1.33 | 1.17 |
| NHL - Nodal | 301 | 244.3 | 1.23* | 1.1 | 1.38 | 0.86 |
| NHL - Extranodal | 141 | 120.78 | 1.17 | 0.98 | 1.38 | 0.31 |
| Myeloma | 130 | 118.73 | 1.09 | 0.91 | 1.3 | 0.17 |
| Leukemia | 317 | 245.64 | 1.29* | 1.15 | 1.44 | 1.09 |
| Lymphocytic Leukemia | 189 | 125.68 | 1.50* | 1.3 | 1.73 | 0.96 |
| Acute Lymphocytic Leukemia | 11 | 8.47 | 1.3 | 0.65 | 2.32 | 0.04 |
| Chronic Lymphocytic Leukemia | 170 | 109.24 | 1.56* | 1.33 | 1.81 | 0.92 |
| Other Lymphocytic Leukemia | 8 | 7.97 | 1 | 0.43 | 1.98 | 0 |
| Non-Lymphocytic Leukemia | 128 | 119.96 | 1.07 | 0.89 | 1.27 | 0.12 |
| Aleukemic, Subleukemic and NOS | 8 | 6.39 | 1.25 | 0.54 | 2.47 | 0.02 |

Abbreviations: CI LB, confidence interval lower bound; CI UB, confidence interval upper bound; E, expected; NHL, non-hodgkin lymphoma; NOS, not otherwise specified; O, observed; SIR, standardized incidence ratio

* P<0.05

^a^Excess absolute risk is per 10,000

| *Supplementary Table 2: Second Primary Malignancies by Specific Site Following First Primary Acral Lentiginous Melanoma* | | | | | | |
| --- | --- | --- | --- | --- | --- | --- |
|  | O | E | O/E | 95% CI LB | 95% CI UB | EAR^a^ |
| All Sites | 235 | 147.45 | 1.59* | 1.4 | 1.81 | 79.56 |
| All Solid Tumors | 210 | 129.95 | 1.62* | 1.4 | 1.85 | 72.75 |
| Oral Cavity and Pharynx | 2 | 3.31 | 0.6 | 0.07 | 2.18 | -1.19 |
| Skin excluding Basal and Squamous | 82 | 7.45 | 11.00* | 8.75 | 13.65 | 67.74 |
| Melanoma of the Skin | 82 | 6.72 | 12.19* | 9.7 | 15.14 | 68.41 |
| Other Non-Epithelial Skin | 0 | 0.73 | 0 | 0 | 5.05 | -0.66 |
| Endocrine System | 9 | 2.55 | 3.53* | 1.61 | 6.7 | 5.86 |
| Thyroid | 9 | 2.4 | 3.74* | 1.71 | 7.11 | 5.99 |
| Thymus, Adrenal Gland and Other Endocrine | 0 | 0.15 | 0 | 0 | 25.04 | -0.13 |
| All Lymphatic and Hematopoietic Diseases | 20 | 13.36 | 1.5 | 0.91 | 2.31 | 6.03 |
| Lymphoma | 14 | 6.79 | 2.06* | 1.13 | 3.46 | 6.55 |
| Hodgkin Lymphoma | 2 | 0.36 | 5.56 | 0.67 | 20.1 | 1.49 |
| Non-Hodgkin Lymphoma | 12 | 6.43 | 1.87 | 0.96 | 3.26 | 5.06 |
| Myeloma | 3 | 2.31 | 1.3 | 0.27 | 3.79 | 0.62 |
| Leukemia | 3 | 4.26 | 0.7 | 0.15 | 2.06 | -1.15 |
| Aleukemic, Subleukemic and NOS | 0 | 0.13 | 0 | 0 | 27.53 | -0.12 |

Abbreviations: CI LB, confidence interval lower bound; CI UB, confidence interval upper bound; E, expected; NOS, not otherwise specified; O, observed; SIR, standardized incidence ratio

* P<0.05

^a^Excess absolute risk is per 10,000

| *Supplementary Table 3: Second Primary Malignancies by Specific Site Following First Primary Mucosal Melanoma* | | | | | | |
| --- | --- | --- | --- | --- | --- | --- |
|  | O | E | O/E | 95% CI LB | 95% CI UB | EAR^a^ |
| All Sites | 203 | 95.01 | 2.14* | 1.85 | 2.45 | 153.59 |
| All Solid Tumors | 191 | 83.54 | 2.29* | 1.97 | 2.63 | 152.84 |
| Floor of Mouth, and Gum and Other Mouth | 4 | 0.46 | 8.73* | 2.38 | 22.36 | 5.04 |
| Floor of Mouth | 1 | 0.1 | 10.06 | 0.25 | 56.08 | 1.28 |
| Gum and Other Mouth | 3 | 0.36 | 8.36* | 1.72 | 24.44 | 3.76 |
| Respiratory System | 31 | 15.11 | 2.05* | 1.39 | 2.91 | 22.6 |
| Nose, Nasal Cavity and Middle Ear | 11 | 0.13 | 82.22* | 41.05 | 147.12 | 15.45 |
| Larynx | 0 | 0.51 | 0 | 0 | 7.26 | -0.72 |
| Pleura | 0 | 0.01 | 0 | 0 | 542.27 | -0.01 |
| Lung, Bronchus, Trachea, Mediastinum and Other Resp Org | 20 | 14.46 | 1.38 | 0.84 | 2.14 | 7.88 |
| Skin excluding Basal and Squamous | 40 | 4.43 | 9.03* | 6.45 | 12.29 | 50.59 |
| Melanoma of the Skin | 40 | 3.98 | 10.05* | 7.18 | 13.68 | 51.23 |
| Other Non-Epithelial Skin | 0 | 0.45 | 0 | 0 | 8.21 | -0.64 |
| Female Genital System | 42 | 6.66 | 6.31* | 4.54 | 8.52 | 50.26 |
| Cervix Uteri | 2 | 0.57 | 3.5 | 0.42 | 12.63 | 2.03 |
| Corpus and Uterus, NOS | 5 | 3.54 | 1.41 | 0.46 | 3.3 | 2.08 |
| Ovary | 0 | 1.81 | 0 | 0 | 2.04 | -2.57 |
| Vagina | 7 | 0.12 | 56.24* | 22.61 | 115.87 | 9.78 |
| Vulva | 27 | 0.45 | 60.60* | 39.94 | 88.18 | 37.77 |
| Other Female Genital Organs | 1 | 0.17 | 5.88 | 0.15 | 32.77 | 1.18 |
| Urinary System | 20 | 7.42 | 2.69* | 1.65 | 4.16 | 17.89 |
| Urinary Bladder | 6 | 4.41 | 1.36 | 0.5 | 2.96 | 2.26 |
| Kidney | 9 | 2.54 | 3.54* | 1.62 | 6.72 | 9.19 |
| Renal Pelvis | 0 | 0.24 | 0 | 0 | 15.25 | -0.34 |
| Ureter | 0 | 0.15 | 0 | 0 | 24.9 | -0.21 |
| Other Urinary Organs | 5 | 0.08 | 65.01* | 21.11 | 151.72 | 7 |
| Endocrine System | 8 | 1.68 | 4.75* | 2.05 | 9.36 | 8.98 |
| Thyroid | 7 | 1.59 | 4.40* | 1.77 | 9.06 | 7.69 |
| Thymus, Adrenal Gland and Other Endocrine | 1 | 0.09 | 10.84 | 0.27 | 60.42 | 1.29 |

Abbreviations: CI LB, confidence interval lower bound; CI UB, confidence interval upper bound; E, expected; NOS, not otherwise specified; O, observed; SIR, standardized incidence ratio

* P<0.05

^a^Excess absolute risk is per 10,000

| *Supplementary Table 4: Second Primary Malignancies by Specific Site Following First Primary Uveal Melanoma* | | | | | | |
| --- | --- | --- | --- | --- | --- | --- |
|  | O | E | O/E | 95% CI LB | 95% CI UB | EAR^a^ |
| All Sites | 586 | 472.5 | 1.24* | 1.14 | 1.34 | 33.04 |
| All Solid Tumors | 522 | 417.4 | 1.25* | 1.15 | 1.36 | 30.47 |
| Skin excluding Basal and Squamous | 73 | 27.14 | 2.69* | 2.11 | 3.38 | 13.35 |
| Melanoma of the Skin | 72 | 24.77 | 2.91* | 2.27 | 3.66 | 13.75 |
| Other Non-Epithelial Skin | 1 | 2.37 | 0.42 | 0.01 | 2.35 | -0.4 |
| Urinary System | 52 | 44.57 | 1.17 | 0.87 | 1.53 | 2.16 |
| Urinary Bladder | 25 | 27.39 | 0.91 | 0.59 | 1.35 | -0.69 |
| Kidney | 25 | 14.86 | 1.68* | 1.09 | 2.48 | 2.95 |
| Renal Pelvis | 1 | 1.16 | 0.86 | 0.02 | 4.81 | -0.05 |
| Ureter | 0 | 0.73 | 0 | 0 | 5.04 | -0.21 |
| Other Urinary Organs | 1 | 0.43 | 2.31 | 0.06 | 12.88 | 0.17 |
| Eye and Orbit | 31 | 0.84 | 36.92* | 25.09 | 52.41 | 8.78 |
| Eye and Orbit - Non-Melanoma | 1 | 0.16 | 6.14 | 0.16 | 34.2 | 0.24 |
| Eye and Orbit - Melanoma | 30 | 0.68 | 44.34* | 29.91 | 63.29 | 8.54 |
| Endocrine System | 28 | 7.85 | 3.57* | 2.37 | 5.15 | 5.87 |
| Thyroid | 28 | 7.4 | 3.79* | 2.52 | 5.47 | 6 |
| Thymus, Adrenal Gland and Other Endocrine | 0 | 0.45 | 0 | 0 | 8.11 | -0.13 |
| Miscellaneous | 22 | 10.02 | 2.20* | 1.38 | 3.33 | 3.49 |

Abbreviations: CI LB, confidence interval lower bound; CI UB, confidence interval upper bound; E, expected; O, observed; SIR, standardized incidence ratio

* P<0.05

^a^Excess absolute risk is per 10,000

| *Supplementary Table 5: Risk of Second Primary Malignancy Distributed by Patient Age* | | | | | | | | | | | | | | | | | | | |
| --- | --- | --- | --- | --- | --- | --- | --- | --- | --- | --- | --- | --- | --- | --- | --- | --- | --- | --- | --- |
|  |  | 0-49 | | | | | | 50-64 | | | | | | 65+ | | | | | |
|  |  | O | E | O/E | 95% CI LB | 95% CI UB | EAR | O | E | O/E | 95% CI LB | 95% CI UB | EAR | O | E | O/E | 95% CI LB | 95% CI UB | EAR^a^ |
| Cutaneous Melanoma | All Sites | 1,136 | 400.19 | 2.84* | 2.68 | 3.01 | 42.03 | 3,437 | 2,066.35 | 1.66* | 1.61 | 1.72 | 60.34 | 7,899 | 5,770.98 | 1.37* | 1.34 | 1.4 | 83.55 |
|  | Tongue | 2 | 3.13 | 0.64 | 0.08 | 2.31 | -0.06 | 21 | 24.47 | 0.86 | 0.53 | 1.31 | -0.15 | 25 | 38.42 | 0.65* | 0.42 | 0.96 | -0.53 |
|  | Salivary Gland | 6 | 1.28 | 4.68* | 1.72 | 10.19 | 0.27 | 4 | 4.92 | 0.81 | 0.22 | 2.08 | -0.04 | 38 | 18.35 | 2.07* | 1.47 | 2.84 | 0.77 |
|  | Floor of Mouth | 0 | 0.42 | 0 | 0 | 8.85 | -0.02 | 0 | 3.87 | 0.00* | 0 | 0.95 | -0.17 | 3 | 6.2 | 0.48 | 0.1 | 1.41 | -0.13 |
|  | Pharynx | 3 | 1.18 | 2.54 | 0.52 | 7.42 | 0.1 | 5 | 9.33 | 0.54 | 0.17 | 1.25 | -0.19 | 6 | 16.49 | 0.36* | 0.13 | 0.79 | -0.41 |
|  | Esophagus | 2 | 1.89 | 1.06 | 0.13 | 3.83 | 0.01 | 13 | 23.12 | 0.56* | 0.3 | 0.96 | -0.45 | 63 | 72.55 | 0.87 | 0.67 | 1.11 | -0.37 |
|  | Stomach | 2 | 4.7 | 0.43 | 0.05 | 1.54 | -0.15 | 19 | 26.54 | 0.72 | 0.43 | 1.12 | -0.33 | 68 | 97.63 | 0.70* | 0.54 | 0.88 | -1.16 |
|  | Colon, Rectum and Anus | 25 | 31.57 | 0.79 | 0.51 | 1.17 | -0.38 | 152 | 184.48 | 0.82* | 0.7 | 0.97 | -1.43 | 482 | 576 | 0.84* | 0.76 | 0.91 | -3.69 |
|  | Colon and Rectum | 20 | 29.28 | 0.68 | 0.42 | 1.06 | -0.53 | 141 | 173.59 | 0.81* | 0.68 | 0.96 | -1.43 | 467 | 559.49 | 0.83* | 0.76 | 0.91 | -3.63 |
|  | Colon excluding Rectum | 12 | 17.4 | 0.69 | 0.36 | 1.2 | -0.31 | 94 | 108.99 | 0.86 | 0.7 | 1.06 | -0.66 | 361 | 422.5 | 0.85* | 0.77 | 0.95 | -2.41 |
|  | Appendix | 2 | 1.71 | 1.17 | 0.14 | 4.22 | 0.02 | 11 | 4.87 | 2.26* | 1.13 | 4.04 | 0.27 | 14 | 6.73 | 2.08* | 1.14 | 3.49 | 0.29 |
|  | Sigmoid Colon | 4 | 6.6 | 0.61 | 0.17 | 1.55 | -0.15 | 33 | 38.38 | 0.86 | 0.59 | 1.21 | -0.24 | 70 | 95.6 | 0.73* | 0.57 | 0.93 | -1.01 |
|  | Large Intestine, NOS | 1 | 0.82 | 1.22 | 0.03 | 6.81 | 0.01 | 2 | 5.42 | 0.37 | 0.04 | 1.33 | -0.15 | 20 | 29.84 | 0.67 | 0.41 | 1.04 | -0.39 |
|  | Rectum and Rectosigmoid Junction | 8 | 11.87 | 0.67 | 0.29 | 1.33 | -0.22 | 47 | 64.59 | 0.73* | 0.53 | 0.97 | -0.77 | 106 | 136.99 | 0.77* | 0.63 | 0.94 | -1.22 |
|  | Rectum | 6 | 9 | 0.67 | 0.24 | 1.45 | -0.17 | 37 | 48.99 | 0.76 | 0.53 | 1.04 | -0.53 | 76 | 101.29 | 0.75* | 0.59 | 0.94 | -0.99 |
|  | Rectum, Rectosig Junct, Anus, Anal Canal and Anorectum | 13 | 14.17 | 0.92 | 0.49 | 1.57 | -0.07 | 58 | 75.49 | 0.77* | 0.58 | 0.99 | -0.77 | 121 | 153.5 | 0.79* | 0.65 | 0.94 | -1.28 |
|  | Liver | 0 | 3.12 | 0 | 0 | 1.18 | -0.18 | 24 | 46.46 | 0.52* | 0.33 | 0.77 | -0.99 | 47 | 78.02 | 0.60* | 0.44 | 0.8 | -1.22 |
|  | Gallbladder | 0 | 0.51 | 0 | 0 | 7.26 | -0.03 | 1 | 3.48 | 0.29 | 0.01 | 1.6 | -0.11 | 3 | 15.28 | 0.20* | 0.04 | 0.57 | -0.48 |
|  | Lung and Bronchus | 32 | 16.96 | 1.89* | 1.29 | 2.66 | 0.86 | 196 | 220.35 | 0.89 | 0.77 | 1.02 | -1.07 | 700 | 928.04 | 0.75* | 0.7 | 0.81 | -8.95 |
|  | Soft Tissue including Heart | 14 | 4.22 | 3.31* | 1.81 | 5.56 | 0.56 | 22 | 12.24 | 1.80* | 1.13 | 2.72 | 0.43 | 70 | 33.67 | 2.08* | 1.62 | 2.63 | 1.43 |
|  | Melanoma of the Skin | 685 | 36.93 | 18.55* | 17.19 | 19.99 | 37.02 | 1,305 | 119.41 | 10.93* | 10.34 | 11.54 | 52.19 | 2,466 | 310.5 | 7.94* | 7.63 | 8.26 | 84.63 |
|  | Other Non-Epithelial Skin | 3 | 1.65 | 1.82 | 0.38 | 5.32 | 0.08 | 16 | 6.13 | 2.61* | 1.49 | 4.24 | 0.43 | 108 | 36.41 | 2.97* | 2.43 | 3.58 | 2.81 |
|  | Cervix Uteri | 8 | 13.37 | 0.6 | 0.26 | 1.18 | -0.31 | 7 | 12.3 | 0.57 | 0.23 | 1.17 | -0.23 | 5 | 9.92 | 0.5 | 0.16 | 1.18 | -0.19 |
|  | Prostate | 27 | 11.07 | 2.44* | 1.61 | 3.55 | 0.91 | 473 | 382.81 | 1.24* | 1.13 | 1.35 | 3.97 | 1,265 | 1,044.68 | 1.21* | 1.15 | 1.28 | 8.65 |
|  | Other Male Genital Organs | 0 | 0.07 | 0 | 0 | 53.04 | 0 | 3 | 0.58 | 5.16* | 1.06 | 15.07 | 0.11 | 4 | 2.05 | 1.95 | 0.53 | 5 | 0.08 |
|  | Urinary Bladder | 4 | 6.13 | 0.65 | 0.18 | 1.67 | -0.12 | 86 | 76.81 | 1.12 | 0.9 | 1.38 | 0.4 | 354 | 430.81 | 0.82* | 0.74 | 0.91 | -3.02 |
|  | Kidney | 22 | 14.49 | 1.52 | 0.95 | 2.3 | 0.43 | 109 | 78.43 | 1.39* | 1.14 | 1.68 | 1.35 | 197 | 172.29 | 1.14 | 0.99 | 1.31 | 0.97 |
|  | Eye and Orbit - Melanoma | 2 | 0.81 | 2.47 | 0.3 | 8.91 | 0.07 | 15 | 3.55 | 4.22* | 2.36 | 6.96 | 0.5 | 12 | 7.5 | 1.6 | 0.83 | 2.8 | 0.18 |
|  | Brain | 14 | 7.7 | 1.82 | 0.99 | 3.05 | 0.36 | 30 | 25.76 | 1.16 | 0.79 | 1.66 | 0.19 | 70 | 55.42 | 1.26 | 0.98 | 1.6 | 0.57 |
|  | Thyroid | 74 | 37.03 | 2.00* | 1.57 | 2.51 | 2.11 | 106 | 54.17 | 1.96* | 1.6 | 2.37 | 2.28 | 80 | 51.26 | 1.56* | 1.24 | 1.94 | 1.13 |
|  | All Lymphatic and Hematopoietic Diseases | 39 | 35.3 | 1.1 | 0.79 | 1.51 | 0.21 | 235 | 159.9 | 1.47* | 1.29 | 1.67 | 3.31 | 638 | 557.37 | 1.14* | 1.06 | 1.24 | 3.17 |
|  | Non-Hodgkin Lymphoma | 21 | 16.88 | 1.24 | 0.77 | 1.9 | 0.24 | 116 | 81.17 | 1.43* | 1.18 | 1.71 | 1.53 | 305 | 267.02 | 1.14* | 1.02 | 1.28 | 1.49 |
|  | NHL - Nodal | 14 | 11.12 | 1.26 | 0.69 | 2.11 | 0.16 | 79 | 55.82 | 1.42* | 1.12 | 1.76 | 1.02 | 208 | 177.35 | 1.17* | 1.02 | 1.34 | 1.2 |
|  | Leukemia | 11 | 10.02 | 1.1 | 0.55 | 1.96 | 0.06 | 81 | 47.89 | 1.69* | 1.34 | 2.1 | 1.46 | 225 | 187.73 | 1.20* | 1.05 | 1.37 | 1.46 |
|  | Lymphocytic Leukemia | 5 | 3.94 | 1.27 | 0.41 | 2.96 | 0.06 | 53 | 26.66 | 1.99* | 1.49 | 2.6 | 1.16 | 131 | 95.08 | 1.38* | 1.15 | 1.63 | 1.41 |
|  | Chronic Lymphocytic Leukemia | 5 | 1.91 | 2.62 | 0.85 | 6.12 | 0.18 | 43 | 21.89 | 1.96* | 1.42 | 2.65 | 0.93 | 122 | 85.44 | 1.43* | 1.19 | 1.7 | 1.44 |
| Acral Lentiginous Melanoma | All Sites | 12 | 5.11 | 2.35* | 1.21 | 4.1 | 31.99 | 51 | 31.27 | 1.63* | 1.21 | 2.14 | 55.48 | 172 | 111.08 | 1.55* | 1.33 | 1.8 | 115.11 |
|  | Appendix | 0 | 0.02 | 0 | 0 | 179.37 | -0.1 | 2 | 0.07 | 27.35* | 3.31 | 98.79 | 5.42 | 1 | 0.13 | 7.75 | 0.2 | 43.16 | 1.65 |
|  | Liver, Gallbladder, Intrahep Bile Duct and Other Biliary | 0 | 0.06 | 0 | 0 | 57.01 | -0.3 | 0 | 0.88 | 0 | 0 | 4.21 | -2.46 | 0 | 2.76 | 0 | 0 | 1.33 | -5.22 |
|  | Peritoneum, Omentum and Mesentery | 0 | 0 | 0 | 0 | 849.2 | -0.02 | 1 | 0.04 | 22.34 | 0.57 | 124.47 | 2.69 | 1 | 0.15 | 6.56 | 0.17 | 36.56 | 1.6 |
|  | Soft Tissue including Heart | 0 | 0.05 | 0 | 0 | 69.88 | -0.25 | 2 | 0.18 | 11.00* | 1.33 | 39.75 | 5.11 | 2 | 0.63 | 3.18 | 0.39 | 11.49 | 2.59 |
|  | Melanoma of the Skin | 6 | 0.4 | 15.02* | 5.51 | 32.69 | 26 | 18 | 1.5 | 12.03* | 7.13 | 19.01 | 46.4 | 58 | 4.83 | 12.01* | 9.12 | 15.53 | 100.46 |
|  | Female Genital System | 0 | 0.52 | 0 | 0 | 7.12 | -2.41 | 0 | 2.54 | 0 | 0 | 1.45 | -7.13 | 2 | 4.81 | 0.42 | 0.05 | 1.5 | -5.3 |
|  | Thyroid | 1 | 0.45 | 2.21 | 0.06 | 12.33 | 2.54 | 2 | 0.9 | 2.23 | 0.27 | 8.05 | 3.1 | 6 | 1.05 | 5.69* | 2.09 | 12.38 | 9.34 |
|  | Lymphoma | 0 | 0.27 | 0 | 0 | 13.59 | -1.26 | 3 | 1.26 | 2.38 | 0.49 | 6.97 | 4.9 | 11 | 5.26 | 2.09* | 1.04 | 3.74 | 10.85 |
| Mucosal Melanoma | All Sites | 16 | 2.81 | 5.70* | 3.26 | 9.26 | 106.8 | 48 | 17 | 2.82* | 2.08 | 3.74 | 162.02 | 139 | 75.21 | 1.85* | 1.55 | 2.18 | 164.32 |
|  | Gum and Other Mouth | 0 | 0.01 | 0 | 0 | 560.38 | -0.05 | 1 | 0.05 | 19.71 | 0.5 | 109.81 | 4.96 | 2 | 0.3 | 6.64 | 0.8 | 23.97 | 4.38 |
|  | Nose, Nasal Cavity and Middle Ear | 1 | 0 | 227.12* | 5.75 | 1,265.45 | 8.06 | 2 | 0.02 | 84.50* | 10.23 | 305.25 | 10.33 | 8 | 0.11 | 75.68* | 32.67 | 149.12 | 20.33 |
|  | Melanoma of the Skin | 6 | 0.23 | 25.75* | 9.45 | 56.05 | 46.68 | 10 | 0.82 | 12.20* | 5.85 | 22.43 | 47.98 | 24 | 2.93 | 8.20* | 5.25 | 12.19 | 54.28 |
|  | Vagina | 1 | 0 | 343.96* | 8.71 | 1,916.43 | 8.07 | 2 | 0.02 | 105.28* | 12.75 | 380.32 | 10.35 | 4 | 0.1 | 39.00* | 10.63 | 99.85 | 10.04 |
|  | Vulva | 1 | 0.01 | 83.07* | 2.1 | 462.85 | 8 | 7 | 0.06 | 119.20* | 47.92 | 245.59 | 36.28 | 19 | 0.37 | 50.70* | 30.52 | 79.17 | 47.97 |
|  | Kidney | 0 | 0.09 | 0 | 0 | 41.32 | -0.72 | 6 | 0.55 | 10.85* | 3.98 | 23.62 | 28.47 | 3 | 1.9 | 1.58 | 0.33 | 4.61 | 2.83 |
|  | Other Urinary Organs | 0 | 0 | 0 | 0 | 6,469.23 | 0 | 0 | 0.01 | 0 | 0 | 503.42 | -0.04 | 5 | 0.07 | 72.45* | 23.53 | 169.08 | 12.7 |
|  | Thyroid | 1 | 0.27 | 3.65 | 0.09 | 20.32 | 5.87 | 3 | 0.5 | 5.98* | 1.23 | 17.46 | 13.06 | 3 | 0.82 | 3.68 | 0.76 | 10.76 | 5.63 |
| Uveal Melanoma | All Sites | 30 | 13.11 | 2.29* | 1.54 | 3.27 | 29.23 | 161 | 117.27 | 1.37* | 1.17 | 1.6 | 34.47 | 395 | 342.16 | 1.15* | 1.04 | 1.27 | 33.28 |
|  | Salivary Gland | 0 | 0.04 | 0 | 0 | 83.7 | -0.08 | 2 | 0.28 | 7.24 | 0.88 | 26.14 | 1.36 | 3 | 1 | 2.99 | 0.62 | 8.73 | 1.26 |
|  | Melanoma of the Skin | 3 | 1.22 | 2.46 | 0.51 | 7.19 | 3.08 | 21 | 6.57 | 3.20* | 1.98 | 4.88 | 11.37 | 48 | 16.98 | 2.83* | 2.08 | 3.75 | 19.54 |
|  | Kidney | 5 | 0.55 | 9.06* | 2.94 | 21.14 | 7.7 | 9 | 4.39 | 2.05 | 0.94 | 3.89 | 3.63 | 11 | 9.92 | 1.11 | 0.55 | 1.98 | 0.68 |
|  | Eye and Orbit - Melanoma | 4 | 0.03 | 140.13* | 38.18 | 358.78 | 6.87 | 13 | 0.2 | 65.18* | 34.71 | 111.47 | 10.09 | 13 | 0.45 | 28.97* | 15.43 | 49.55 | 7.9 |
|  | Thyroid | 4 | 1.07 | 3.73* | 1.02 | 9.55 | 5.07 | 12 | 2.97 | 4.04* | 2.09 | 7.06 | 7.12 | 12 | 3.35 | 3.58* | 1.85 | 6.25 | 5.45 |
|  | Miscellaneous | 0 | 0.13 | 0 | 0 | 27.51 | -0.23 | 6 | 1.67 | 3.59* | 1.32 | 7.82 | 3.41 | 16 | 8.21 | 1.95* | 1.11 | 3.16 | 4.9 |

Abbreviations: CI LB, confidence interval lower bound; CI UB, confidence interval upper bound; E, expected; NHL, non-hodgkin lymphoma; NOS, not otherwise specified; O, observed; SIR, standardized incidence ratio

* P<0.05

^a^Excess absolute risk is per 10,000
